# Bis{2-[4-(methyl­sulfan­yl)phen­yl]-1*H*-benzimidazol-3-ium} tetra­bromido­cadmate(II) ethanol monosolvate

**DOI:** 10.1107/S1600536811018058

**Published:** 2011-05-20

**Authors:** M. N. Manjunatha, Mohamed Ziaulla, Noor Shahina Begum, K. R. Nagasundara

**Affiliations:** a Department of Chemistry, Bangalore University, Bangalore 560 001, Karnataka, India

## Abstract

In the anion of the title compound, (C_14_H_13_N_2_S)_2_[CdBr_4_]·C_2_H_5_OH, the Cd^II^ atom is in a distorted tetra­hedral environment and one of the Br atoms is disordered over three sites with site-occupancy factors of 0.828 (5), 0.106 (3) and 0.068 (4). In the crystal, inter­molecular N—H⋯O, C—H⋯O and N—H⋯Br inter­actions result in a two-dimensional polymeric network extending parallel to (010).

## Related literature

For general background to benzimidazole derivatives, see: Huang & Scarborough (1999 ▶); Preston (1974 ▶); Zarrinmayeh *et al.* (1998 ▶); Zhu *et al.* (2000 ▶). For related structures, see: Ziaulla *et al.* (2011 ▶). For hydrogen bonding, see: Bernstein *et al.* (1995 ▶); Nardelli (1983 ▶).
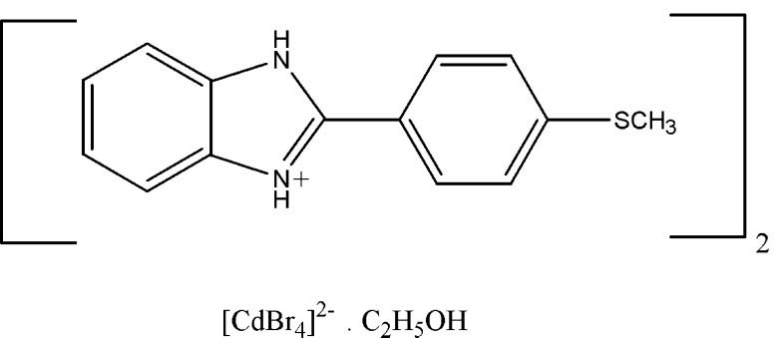


## Experimental

### 

#### Crystal data

(C_14_H_13_N_2_S)_2_[CdBr_4_]·C_2_H_6_O
*M* *_r_* = 960.76
Orthorhombic, 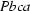

*a* = 22.1321 (15) Å
*b* = 13.8746 (10) Å
*c* = 22.2594 (16) Å
*V* = 6835.3 (8) Å^3^
*Z* = 8
Mo *K*α radiation
μ = 5.47 mm^−1^
*T* = 123 K
0.20 × 0.18 × 0.18 mm

#### Data collection

Bruker SMART APEX CCD detector diffractometer
Absorption correction: multi-scan (*SADABS*; Sheldrick, 1996 ▶) *T*_min_ = 0.408, *T*_max_ = 0.439
93968 measured reflections
7467 independent reflections
5951 reflections with *I* > 2σ(*I*)
*R*_int_ = 0.084

#### Refinement

*R*[*F* ^2^ > 2σ(*F* ^2^)] = 0.034
*wR*(*F* ^2^) = 0.079
*S* = 0.79
7467 reflections
407 parameters
H atoms treated by a mixture of independent and constrained refinement
Δρ_max_ = 0.87 e Å^−3^
Δρ_min_ = −0.69 e Å^−3^

### 

Data collection: *SMART* (Bruker, 1998 ▶); cell refinement: *SAINT-Plus* (Bruker, 1998 ▶); data reduction: *SAINT-Plus*; program(s) used to solve structure: *SHELXS97* (Sheldrick, 2008 ▶); program(s) used to refine structure: *SHELXL97* (Sheldrick, 2008 ▶); molecular graphics: *ORTEP-3* (Farrugia, 1997 ▶) and *CAMERON* (Watkin *et al.*, 1996) ▶; software used to prepare material for publication: *WinGX* (Farrugia, 1999 ▶).

## Supplementary Material

Crystal structure: contains datablocks global, I. DOI: 10.1107/S1600536811018058/ds2093sup1.cif

Structure factors: contains datablocks I. DOI: 10.1107/S1600536811018058/ds2093Isup2.hkl

Additional supplementary materials:  crystallographic information; 3D view; checkCIF report

## Figures and Tables

**Table 1:** Hydrogen-bond geometry (Å, °)

*D*—H⋯*A*	*D*—H	H⋯*A*	*D*⋯*A*	*D*—H⋯*A*
N1—H1*N*⋯Br3	0.79 (7)	2.51 (7)	3.272 (5)	164 (5)
N2—H2*N*⋯Br2^i^	0.81 (7)	2.50 (7)	3.267 (4)	160 (5)
N4—H4*N*⋯O1^ii^	0.83 (7)	1.88 (7)	2.679 (6)	161 (6)
C4—H4⋯O1^ii^	0.95	2.55	3.464 (8)	160

Symmetry codes: (i) 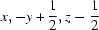; (ii) 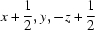.

**Fig. 1.**
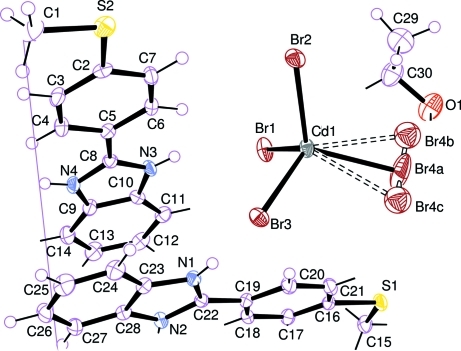
ORTEP (Farrugia, 1997) view of the title compound, showing 50% probability ellipsoids and the atom numbering scheme.

**Fig. 2.**
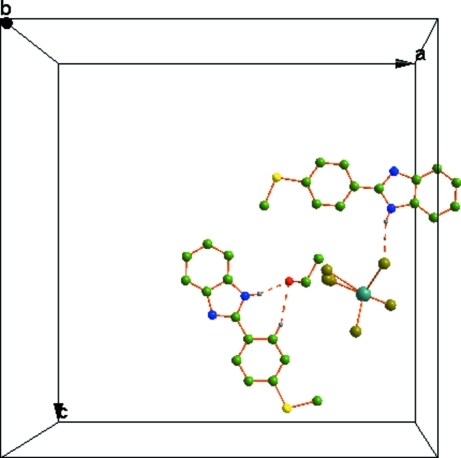
A unit cell packing of the title compound showing intermolecular interactions with dotted lines. H-atoms not involved in hydrogen bonding have been excluded.

## supplementary crystallographic information

### Comment

Benzimidazole derivatives are effective against the human cytomegalo virus (HCMV) (Zhu *et al.*, 2000) and are also efficient selective neuropeptide Y Y1 receptor antagonists (Zarrinmayeh *et al.*, 1998). In addition, benzimidazole derivatives exhibit a number of important pharmacological properties, such as antihistaminic, anti-ulcerative, antiallergic and antipyretic. The described methods for the synthesis of benzimidazoles make use of solid-phase synthesis *via o*-nitroanilines (Preston *et al.*, 1974; Huang *et al.*, 1999) or the condensation of o-phenylenediamines with carboxylic acid derivatives, aldehydes and aryl halides. The benzimidazole derivative has been used as a ligand for complexation with cadmium metal to give the above metal complex. In the title compound, as shown in Fig. 1, there are two cation,one tetrabormocadmate(II) anion and an ethanol molecule in the asymmetric unit. One of the coordinated bromine atom Br4 of the anion is disordered over three sites (Br4A/Br4B/Br4C) with site occupancy factors 0.83, 0,11 and 0.06 resulting in one major and two minor components.The Cd^II^ atom has a distorted tetrahedral geometry, coordinating with four terminal bromine atoms with the bond lengths in the range 2.5616 (7)Å to 2.6177 (6) Å. The Br—Cd—Br bond angles are between 111.37 (3)° and 107.14 (2)°, The benzimidazole and thiomethyl phenyl rings are virtually planar and inclined at an dihedral angle 5.19 (2)°. The molecular structure is primarly stablised by intramolecular N—H···Br interactions. The bond lengths and angles for the benzimidazole moiety of the molecule are in good agreement, within experimental errors, with those observed in other benzimidazole derivatives (Ziaulla *et al.*, 2011). Further, the crystal structure is stabilized by intermolecular N—H···O, C—H···O and N—H···Br hydrogen bonds.

### Experimental

An ethanolic solution (15 ml) of the 2-(4-Methyl sulfanyl phenyl)-1*H*- benzimidazole) (0.960 mg, 2 mmol) was added to a solution of cadmium(II) bromide (0.272 mg, 1 mmol) in ethanol (25 ml). The mixture was then treated with 48% HBr (2–3 ml) followed by liquid Br_2_ (2–3 ml). The mixture was refluxed for nearly six hours during which yellow crystals suitable for X-ray analysis were obtained. The crystals were washed with cold ethanol and dried in vacuum over P_2_O_5_. (yield 1.23 mg, 85%).

### Refinement

The H atoms were placed at calculated positions in the riding model approximation with N—H = 0.83 and C—H = 0.95 Å, and *U*_iso_(H) = 1.2*U*_eq_(N/C).

### Crystal data

**Table:**

(C_14_H_13_N_2_S)_2_[CdBr_4_]·C_2_H_6_O	*F*(000) = 3744
*M**_r_* = 960.76	*D*_x_ = 1.867 Mg m^−^^3^
Orthorhombic, *P**b**c**a*	Mo *K*α radiation, λ = 0.71073 Å
Hall symbol: -P 2ac 2ab	Cell parameters from 7467 reflections
*a* = 22.1321 (15) Å	θ = 1.8–27.0°
*b* = 13.8746 (10) Å	µ = 5.47 mm^−^^1^
*c* = 22.2594 (16) Å	*T* = 123 K
*V* = 6835.3 (8) Å^3^	Block, yellow
*Z* = 8	0.20 × 0.18 × 0.18 mm

### Data collection

**Table:**

Bruker SMART APEX CCD detector diffractometer	7467 independent reflections
Radiation source: Enhance (Mo) X-ray Source	5951 reflections with *I* > 2σ(*I*)
graphite	*R*_int_ = 0.084
ω scans	θ_max_ = 27.0°, θ_min_ = 1.8°
Absorption correction: multi-scan (*SADABS*; Sheldrick, 1996)	*h* = −28→28
*T*_min_ = 0.408, *T*_max_ = 0.439	*k* = −17→17
93968 measured reflections	*l* = −28→28

### Refinement

**Table:**

Refinement on *F*^2^	Primary atom site location: structure-invariant direct methods
Least-squares matrix: full	Secondary atom site location: difference Fourier map
*R*[*F*^2^ > 2σ(*F*^2^)] = 0.034	Hydrogen site location: inferred from neighbouring sites
*wR*(*F*^2^) = 0.079	H atoms treated by a mixture of independent and constrained refinement
*S* = 0.79	*w* = 1/[σ^2^(*F*_o_^2^) + (0.0318*P*)^2^ + 49.2262*P*] where *P* = (*F*_o_^2^ + 2*F*_c_^2^)/3
7467 reflections	(Δ/σ)_max_ = 0.001
407 parameters	Δρ_max_ = 0.87 e Å^−^^3^
0 restraints	Δρ_min_ = −0.69 e Å^−^^3^

### Special details

**Table:**

Geometry. All s.u.'s (except the s.u. in the dihedral angle between two l.s. planes) are estimated using the full covariance matrix. The cell s.u.'s are taken into account individually in the estimation of s.u.'s in distances, angles and torsion angles; correlations between s.u.'s in cell parameters are only used when they are defined by crystal symmetry. An approximate (isotropic) treatment of cell s.u.'s is used for estimating s.u.'s involving l.s. planes.
Refinement. Refinement of *F*^2^ against ALL reflections. The weighted *R*-factor *wR* and goodness of fit *S* are based on *F*^2^, conventional *R*-factors *R* are based on *F*, with *F* set to zero for negative *F*^2^. The threshold expression of *F*^2^ > 2σ(*F*^2^) is used only for calculating *R*-factors(gt) *etc*. and is not relevant to the choice of reflections for refinement. *R*-factors based on *F*^2^ are statistically about twice as large as those based on *F*, and *R*- factors based on ALL data will be even larger.

### Fractional atomic coordinates and isotropic or equivalent isotropic displacement parameters (Å^2^)

**Table:**

	*x*	*y*	*z*	*U*_iso_*/*U*_eq_	Occ. (<1)
Cd1	0.348496 (11)	0.21808 (2)	0.361336 (13)	0.02080 (7)	
Br1	0.326079 (18)	0.11642 (3)	0.26634 (2)	0.02983 (11)	
Br2	0.399388 (19)	0.11298 (3)	0.441935 (19)	0.02699 (10)	
Br3	0.425609 (17)	0.35205 (3)	0.330092 (18)	0.02342 (9)	
Br4A	0.25223 (3)	0.29865 (10)	0.40079 (6)	0.0445 (4)	0.828 (5)
Br4B	0.2514 (2)	0.2592 (6)	0.4250 (3)	0.0302 (17)*	0.106 (3)
Br4C	0.2621 (4)	0.3426 (10)	0.3968 (3)	0.028 (3)*	0.068 (4)
S1	0.11862 (4)	0.36770 (8)	0.16669 (5)	0.0261 (2)	
S2	0.64644 (5)	0.09455 (8)	0.43078 (5)	0.0259 (2)	
N1	0.42680 (14)	0.3486 (2)	0.18313 (16)	0.0184 (7)	
H1N	0.4190 (18)	0.346 (3)	0.217 (2)	0.011 (11)*	
N2	0.41912 (14)	0.3569 (2)	0.08615 (16)	0.0172 (7)	
H2N	0.405 (2)	0.362 (3)	0.051 (2)	0.022 (12)*	
N3	0.45281 (14)	0.1032 (2)	0.19059 (16)	0.0186 (7)	
H3N	0.429 (2)	0.100 (4)	0.218 (2)	0.038 (15)*	
N4	0.53761 (15)	0.1058 (2)	0.14156 (16)	0.0208 (7)	
H4N	0.575 (2)	0.103 (3)	0.136 (2)	0.034 (13)*	
O1	0.15209 (14)	0.1207 (3)	0.39651 (19)	0.0460 (9)	
H1	0.1697	0.1741	0.3994	0.069*	
C1	0.72537 (18)	0.1017 (3)	0.4124 (2)	0.0306 (10)	
H1A	0.7375	0.0434	0.3907	0.046*	
H1B	0.7491	0.1073	0.4494	0.046*	
H1C	0.7326	0.1583	0.3871	0.046*	
C2	0.61084 (17)	0.0962 (3)	0.36033 (19)	0.0194 (8)	
C3	0.64147 (17)	0.0996 (3)	0.3056 (2)	0.0229 (9)	
H3	0.6844	0.1015	0.3049	0.027*	
C4	0.60955 (16)	0.1002 (3)	0.25272 (19)	0.0214 (8)	
H4	0.6307	0.1015	0.2156	0.026*	
C5	0.54640 (17)	0.0990 (3)	0.25268 (18)	0.0184 (8)	
C6	0.51621 (16)	0.0957 (3)	0.30770 (18)	0.0196 (8)	
H6	0.4733	0.0944	0.3084	0.024*	
C7	0.54755 (17)	0.0943 (3)	0.36072 (19)	0.0212 (8)	
H7	0.5264	0.0920	0.3978	0.025*	
C8	0.51336 (16)	0.1021 (3)	0.19612 (18)	0.0181 (8)	
C9	0.49220 (16)	0.1105 (3)	0.09894 (18)	0.0205 (8)	
C10	0.43776 (17)	0.1091 (3)	0.13000 (18)	0.0186 (8)	
C11	0.38240 (17)	0.1154 (3)	0.1008 (2)	0.0234 (9)	
H11	0.3452	0.1145	0.1222	0.028*	
C12	0.38445 (18)	0.1230 (3)	0.0395 (2)	0.0252 (9)	
H12	0.3476	0.1283	0.0179	0.030*	
C13	0.43935 (19)	0.1233 (3)	0.0071 (2)	0.0279 (9)	
H13	0.4386	0.1280	−0.0354	0.034*	
C14	0.49412 (19)	0.1170 (3)	0.0366 (2)	0.0271 (9)	
H14	0.5313	0.1170	0.0154	0.033*	
C15	0.08636 (17)	0.3561 (3)	0.0930 (2)	0.0263 (9)	
H15A	0.1001	0.4096	0.0677	0.039*	
H15B	0.0422	0.3575	0.0959	0.039*	
H15C	0.0992	0.2949	0.0751	0.039*	
C16	0.19661 (16)	0.3623 (3)	0.15389 (19)	0.0209 (8)	
C17	0.22338 (16)	0.3494 (3)	0.09802 (19)	0.0203 (8)	
H17	0.1987	0.3427	0.0633	0.024*	
C18	0.28564 (16)	0.3464 (3)	0.09239 (18)	0.0197 (8)	
H18	0.3034	0.3379	0.0539	0.024*	
C19	0.32244 (16)	0.3556 (3)	0.14304 (18)	0.0182 (8)	
C20	0.29562 (17)	0.3688 (3)	0.19946 (19)	0.0233 (8)	
H20	0.3202	0.3754	0.2342	0.028*	
C21	0.23346 (18)	0.3722 (3)	0.2046 (2)	0.0253 (9)	
H21	0.2155	0.3813	0.2430	0.030*	
C22	0.38773 (16)	0.3542 (3)	0.13739 (17)	0.0172 (7)	
C23	0.48535 (16)	0.3490 (3)	0.16087 (18)	0.0180 (8)	
C24	0.54085 (17)	0.3457 (3)	0.1900 (2)	0.0253 (9)	
H24	0.5440	0.3420	0.2325	0.030*	
C25	0.59109 (18)	0.3483 (3)	0.1532 (2)	0.0301 (10)	
H25	0.6301	0.3463	0.1710	0.036*	
C26	0.58653 (17)	0.3537 (3)	0.0909 (2)	0.0273 (9)	
H26	0.6225	0.3555	0.0676	0.033*	
C27	0.53125 (17)	0.3566 (3)	0.0619 (2)	0.0235 (9)	
H27	0.5280	0.3602	0.0194	0.028*	
C28	0.48085 (16)	0.3539 (3)	0.09907 (18)	0.0174 (7)	
C29	0.22850 (16)	0.0414 (3)	0.45435 (18)	0.0460 (13)	
H29A	0.2535	0.0992	0.4589	0.069*	
H29B	0.2542	−0.0160	0.4559	0.069*	
H29C	0.1988	0.0388	0.4869	0.069*	
C30	0.19621 (16)	0.0449 (3)	0.39490 (18)	0.0428 (12)	
H30A	0.2256	0.0570	0.3622	0.051*	
H30B	0.1761	−0.0176	0.3871	0.051*	

### Atomic displacement parameters (Å^2^)

**Table:**

	*U*^11^	*U*^22^	*U*^33^	*U*^12^	*U*^13^	*U*^23^
Cd1	0.01375 (12)	0.02995 (15)	0.01871 (15)	0.00150 (11)	−0.00061 (11)	−0.00047 (12)
Br1	0.01902 (18)	0.0486 (3)	0.0218 (2)	−0.00511 (18)	0.00033 (16)	−0.00842 (19)
Br2	0.0268 (2)	0.0371 (2)	0.0170 (2)	0.00091 (17)	−0.00239 (16)	0.00446 (17)
Br3	0.02397 (19)	0.0292 (2)	0.0171 (2)	−0.00377 (16)	−0.00242 (16)	0.00177 (16)
Br4A	0.0152 (3)	0.0288 (7)	0.0897 (8)	0.0002 (3)	0.0138 (3)	−0.0106 (5)
S1	0.0164 (4)	0.0349 (6)	0.0269 (6)	0.0037 (4)	0.0056 (4)	0.0071 (5)
S2	0.0231 (5)	0.0303 (5)	0.0241 (6)	0.0015 (4)	−0.0071 (4)	−0.0005 (4)
N1	0.0197 (16)	0.0236 (17)	0.0121 (18)	0.0033 (13)	−0.0002 (13)	0.0013 (14)
N2	0.0143 (14)	0.0235 (16)	0.0137 (18)	−0.0019 (12)	−0.0029 (13)	−0.0010 (13)
N3	0.0138 (15)	0.0223 (17)	0.0196 (19)	−0.0024 (12)	0.0015 (13)	0.0029 (14)
N4	0.0147 (15)	0.0261 (17)	0.0218 (19)	0.0031 (13)	0.0018 (13)	0.0018 (14)
O1	0.0256 (16)	0.052 (2)	0.060 (3)	0.0025 (15)	−0.0133 (17)	−0.010 (2)
C1	0.0209 (19)	0.031 (2)	0.040 (3)	0.0003 (17)	−0.0128 (19)	0.002 (2)
C2	0.0216 (18)	0.0139 (17)	0.023 (2)	0.0013 (14)	−0.0059 (16)	0.0002 (15)
C3	0.0143 (17)	0.024 (2)	0.030 (2)	0.0039 (15)	0.0001 (16)	0.0005 (17)
C4	0.0147 (17)	0.027 (2)	0.023 (2)	0.0007 (15)	0.0014 (15)	−0.0019 (17)
C5	0.0180 (17)	0.0178 (18)	0.019 (2)	0.0018 (14)	0.0005 (15)	0.0007 (15)
C6	0.0149 (17)	0.0217 (19)	0.022 (2)	0.0015 (14)	0.0024 (15)	−0.0014 (16)
C7	0.0180 (17)	0.0232 (19)	0.022 (2)	0.0003 (15)	0.0050 (16)	−0.0001 (16)
C8	0.0168 (17)	0.0167 (18)	0.021 (2)	−0.0006 (14)	0.0010 (15)	0.0025 (16)
C9	0.0180 (18)	0.0240 (19)	0.020 (2)	0.0044 (15)	−0.0004 (15)	−0.0003 (16)
C10	0.0219 (18)	0.0176 (18)	0.016 (2)	−0.0021 (14)	0.0003 (15)	0.0011 (15)
C11	0.0181 (18)	0.0229 (19)	0.029 (2)	−0.0014 (15)	−0.0034 (16)	−0.0016 (17)
C12	0.026 (2)	0.024 (2)	0.026 (2)	0.0024 (16)	−0.0112 (17)	−0.0067 (17)
C13	0.035 (2)	0.033 (2)	0.015 (2)	0.0056 (18)	−0.0035 (17)	−0.0049 (18)
C14	0.027 (2)	0.031 (2)	0.023 (2)	0.0022 (17)	0.0060 (17)	−0.0015 (18)
C15	0.0160 (18)	0.034 (2)	0.029 (2)	0.0018 (16)	0.0002 (16)	0.0008 (19)
C16	0.0153 (17)	0.0181 (18)	0.029 (2)	0.0021 (14)	0.0042 (16)	0.0044 (16)
C17	0.0163 (17)	0.0217 (19)	0.023 (2)	−0.0010 (14)	−0.0014 (16)	−0.0011 (16)
C18	0.0169 (17)	0.0224 (19)	0.020 (2)	−0.0003 (14)	0.0039 (15)	0.0006 (16)
C19	0.0182 (17)	0.0171 (17)	0.019 (2)	0.0020 (14)	0.0016 (15)	0.0000 (15)
C20	0.0213 (19)	0.028 (2)	0.021 (2)	0.0022 (16)	0.0011 (16)	0.0035 (17)
C21	0.0239 (19)	0.032 (2)	0.020 (2)	0.0040 (16)	0.0048 (17)	0.0045 (18)
C22	0.0224 (18)	0.0138 (17)	0.015 (2)	−0.0001 (14)	0.0015 (15)	−0.0008 (15)
C23	0.0163 (17)	0.0182 (18)	0.019 (2)	−0.0007 (14)	−0.0027 (15)	−0.0016 (16)
C24	0.0214 (19)	0.031 (2)	0.024 (2)	0.0006 (16)	−0.0071 (17)	0.0011 (18)
C25	0.0187 (19)	0.035 (2)	0.036 (3)	−0.0032 (17)	−0.0103 (18)	0.000 (2)
C26	0.0157 (18)	0.038 (2)	0.028 (2)	0.0009 (16)	0.0009 (16)	0.0081 (19)
C27	0.0196 (18)	0.027 (2)	0.025 (2)	0.0007 (15)	0.0017 (16)	0.0024 (17)
C28	0.0163 (17)	0.0173 (17)	0.019 (2)	−0.0005 (14)	−0.0048 (15)	−0.0011 (15)
C29	0.048 (3)	0.054 (3)	0.036 (3)	−0.005 (3)	−0.006 (2)	−0.003 (3)
C30	0.031 (2)	0.049 (3)	0.049 (3)	0.007 (2)	−0.007 (2)	−0.009 (3)

### Geometric parameters (Å, °)

**Table:**

Cd1—Br4A	2.5612 (6)	C9—C14	1.390 (6)
Cd1—Br2	2.5717 (5)	C9—C10	1.389 (5)
Cd1—Br1	2.5898 (5)	C10—C11	1.390 (5)
Cd1—Br3	2.6175 (5)	C11—C12	1.370 (6)
Cd1—Br4B	2.636 (5)	C11—H11	0.9500
Cd1—Br4C	2.695 (8)	C12—C13	1.413 (6)
Br4A—Br4C	0.654 (14)	C12—H12	0.9500
Br4A—Br4B	0.768 (8)	C13—C14	1.382 (6)
Br4B—Br4C	1.338 (16)	C13—H13	0.9500
S1—C16	1.751 (4)	C14—H14	0.9500
S1—C15	1.797 (4)	C15—H15A	0.9800
S2—C2	1.755 (4)	C15—H15B	0.9800
S2—C1	1.797 (4)	C15—H15C	0.9800
N1—C22	1.338 (5)	C16—C17	1.389 (6)
N1—C23	1.387 (5)	C16—C21	1.400 (6)
N1—H1N	0.77 (4)	C17—C18	1.384 (5)
N2—C22	1.336 (5)	C17—H17	0.9500
N2—C28	1.397 (4)	C18—C19	1.397 (5)
N2—H2N	0.84 (5)	C18—H18	0.9500
N3—C8	1.346 (5)	C19—C20	1.401 (6)
N3—C10	1.392 (5)	C19—C22	1.451 (5)
N3—H3N	0.81 (5)	C20—C21	1.381 (5)
N4—C8	1.329 (5)	C20—H20	0.9500
N4—C9	1.384 (5)	C21—H21	0.9500
N4—H4N	0.84 (5)	C23—C28	1.381 (5)
O1—C30	1.435 (5)	C23—C24	1.390 (5)
O1—H1	0.8400	C24—C25	1.382 (6)
C1—H1A	0.9800	C24—H24	0.9500
C1—H1B	0.9800	C25—C26	1.392 (6)
C1—H1C	0.9800	C25—H25	0.9500
C2—C3	1.395 (6)	C26—C27	1.384 (5)
C2—C7	1.401 (5)	C26—H26	0.9500
C3—C4	1.373 (6)	C27—C28	1.390 (5)
C3—H3	0.9500	C27—H27	0.9500
C4—C5	1.398 (5)	C29—C30	1.5047
C4—H4	0.9500	C29—H29A	0.9800
C5—C6	1.396 (5)	C29—H29B	0.9800
C5—C8	1.457 (5)	C29—H29C	0.9800
C6—C7	1.369 (6)	C30—H30A	0.9900
C6—H6	0.9500	C30—H30B	0.9900
C7—H7	0.9500		
			
Br4A—Cd1—Br2	111.87 (4)	C11—C10—N3	131.9 (4)
Br4A—Cd1—Br1	111.00 (2)	C12—C11—C10	116.2 (4)
Br2—Cd1—Br1	110.162 (19)	C12—C11—H11	121.9
Br4A—Cd1—Br3	108.88 (4)	C10—C11—H11	121.9
Br2—Cd1—Br3	107.605 (16)	C11—C12—C13	122.5 (4)
Br1—Cd1—Br3	107.142 (17)	C11—C12—H12	118.8
Br4A—Cd1—Br4B	16.92 (16)	C13—C12—H12	118.8
Br2—Cd1—Br4B	96.01 (16)	C14—C13—C12	120.8 (4)
Br1—Cd1—Br4B	113.60 (11)	C14—C13—H13	119.6
Br3—Cd1—Br4B	121.39 (17)	C12—C13—H13	119.6
Br4A—Cd1—Br4C	14.0 (3)	C13—C14—C9	116.8 (4)
Br2—Cd1—Br4C	118.01 (18)	C13—C14—H14	121.6
Br1—Cd1—Br4C	116.9 (2)	C9—C14—H14	121.6
Br3—Cd1—Br4C	94.9 (3)	S1—C15—H15A	109.5
Br4B—Cd1—Br4C	29.0 (3)	S1—C15—H15B	109.5
Br4C—Br4A—Br4B	140.2 (8)	H15A—C15—H15B	109.5
Br4C—Br4A—Cd1	94.7 (6)	S1—C15—H15C	109.5
Br4B—Br4A—Cd1	87.1 (4)	H15A—C15—H15C	109.5
Br4A—Br4B—Br4C	18.3 (5)	H15B—C15—H15C	109.5
Br4A—Br4B—Cd1	76.0 (4)	C17—C16—C21	119.1 (3)
Br4C—Br4B—Cd1	77.9 (4)	C17—C16—S1	124.9 (3)
Br4A—Br4C—Br4B	21.6 (5)	C21—C16—S1	116.0 (3)
Br4A—Br4C—Cd1	71.3 (6)	C18—C17—C16	120.6 (4)
Br4B—Br4C—Cd1	73.0 (5)	C18—C17—H17	119.7
C16—S1—C15	103.9 (2)	C16—C17—H17	119.7
C2—S2—C1	103.4 (2)	C17—C18—C19	120.3 (4)
C22—N1—C23	109.4 (3)	C17—C18—H18	119.9
C22—N1—H1N	127 (3)	C19—C18—H18	119.9
C23—N1—H1N	124 (3)	C18—C19—C20	119.2 (3)
C22—N2—C28	109.4 (3)	C18—C19—C22	120.6 (4)
C22—N2—H2N	127 (3)	C20—C19—C22	120.1 (4)
C28—N2—H2N	124 (3)	C21—C20—C19	120.1 (4)
C8—N3—C10	109.1 (3)	C21—C20—H20	120.0
C8—N3—H3N	126 (4)	C19—C20—H20	120.0
C10—N3—H3N	125 (4)	C20—C21—C16	120.6 (4)
C8—N4—C9	109.6 (3)	C20—C21—H21	119.7
C8—N4—H4N	122 (3)	C16—C21—H21	119.7
C9—N4—H4N	128 (3)	N2—C22—N1	108.4 (3)
C30—O1—H1	109.5	N2—C22—C19	126.3 (4)
S2—C1—H1A	109.5	N1—C22—C19	125.3 (4)
S2—C1—H1B	109.5	C28—C23—N1	106.8 (3)
H1A—C1—H1B	109.5	C28—C23—C24	122.0 (4)
S2—C1—H1C	109.5	N1—C23—C24	131.2 (4)
H1A—C1—H1C	109.5	C25—C24—C23	115.7 (4)
H1B—C1—H1C	109.5	C25—C24—H24	122.1
C3—C2—C7	119.5 (4)	C23—C24—H24	122.1
C3—C2—S2	124.2 (3)	C24—C25—C26	122.3 (4)
C7—C2—S2	116.3 (3)	C24—C25—H25	118.9
C4—C3—C2	119.9 (3)	C26—C25—H25	118.9
C4—C3—H3	120.0	C27—C26—C25	122.0 (4)
C2—C3—H3	120.0	C27—C26—H26	119.0
C3—C4—C5	121.0 (4)	C25—C26—H26	119.0
C3—C4—H4	119.5	C28—C27—C26	115.5 (4)
C5—C4—H4	119.5	C28—C27—H27	122.2
C6—C5—C4	118.6 (4)	C26—C27—H27	122.2
C6—C5—C8	121.3 (3)	C23—C28—C27	122.5 (3)
C4—C5—C8	120.1 (4)	C23—C28—N2	106.1 (3)
C7—C6—C5	120.9 (3)	C27—C28—N2	131.4 (4)
C7—C6—H6	119.5	C30—C29—H29A	109.5
C5—C6—H6	119.5	C30—C29—H29B	109.5
C6—C7—C2	120.1 (4)	H29A—C29—H29B	109.5
C6—C7—H7	120.0	C30—C29—H29C	109.5
C2—C7—H7	120.0	H29A—C29—H29C	109.5
N4—C8—N3	108.6 (3)	H29B—C29—H29C	109.5
N4—C8—C5	126.0 (3)	O1—C30—C29	108.9 (2)
N3—C8—C5	125.4 (4)	O1—C30—H30A	109.9
N4—C9—C14	131.7 (4)	C29—C30—H30A	109.9
N4—C9—C10	106.7 (3)	O1—C30—H30B	109.9
C14—C9—C10	121.6 (4)	C29—C30—H30B	109.9
C9—C10—C11	122.1 (4)	H30A—C30—H30B	108.3
C9—C10—N3	106.0 (3)		
			
Br2—Cd1—Br4A—Br4C	118.8 (7)	C8—N4—C9—C14	178.9 (4)
Br1—Cd1—Br4A—Br4C	−117.7 (7)	C8—N4—C9—C10	−0.2 (4)
Br3—Cd1—Br4A—Br4C	0.0 (7)	N4—C9—C10—C11	178.3 (3)
Br4B—Cd1—Br4A—Br4C	140.1 (8)	C14—C9—C10—C11	−1.0 (6)
Br2—Cd1—Br4A—Br4B	−21.3 (4)	N4—C9—C10—N3	−0.2 (4)
Br1—Cd1—Br4A—Br4B	102.2 (4)	C14—C9—C10—N3	−179.4 (4)
Br3—Cd1—Br4A—Br4B	−140.1 (4)	C8—N3—C10—C9	0.6 (4)
Br4C—Cd1—Br4A—Br4B	−140.1 (8)	C8—N3—C10—C11	−177.7 (4)
Cd1—Br4A—Br4B—Br4C	93.9 (10)	C9—C10—C11—C12	0.1 (6)
Br4C—Br4A—Br4B—Cd1	−93.9 (10)	N3—C10—C11—C12	178.1 (4)
Br2—Cd1—Br4B—Br4A	160.2 (4)	C10—C11—C12—C13	0.8 (6)
Br1—Cd1—Br4B—Br4A	−84.7 (4)	C11—C12—C13—C14	−0.8 (6)
Br3—Cd1—Br4B—Br4A	45.3 (4)	C12—C13—C14—C9	−0.1 (6)
Br4C—Cd1—Br4B—Br4A	18.6 (5)	N4—C9—C14—C13	−178.1 (4)
Br4A—Cd1—Br4B—Br4C	−18.6 (5)	C10—C9—C14—C13	0.9 (6)
Br2—Cd1—Br4B—Br4C	141.6 (4)	C15—S1—C16—C17	−1.4 (4)
Br1—Cd1—Br4B—Br4C	−103.3 (4)	C15—S1—C16—C21	178.5 (3)
Br3—Cd1—Br4B—Br4C	26.7 (5)	C21—C16—C17—C18	0.0 (6)
Cd1—Br4A—Br4C—Br4B	−91.1 (9)	S1—C16—C17—C18	179.9 (3)
Br4B—Br4A—Br4C—Cd1	91.1 (9)	C16—C17—C18—C19	0.4 (6)
Cd1—Br4B—Br4C—Br4A	81.9 (10)	C17—C18—C19—C20	−0.6 (6)
Br4A—Br4B—Br4C—Cd1	−81.9 (10)	C17—C18—C19—C22	−178.8 (3)
Br2—Cd1—Br4C—Br4A	−67.1 (7)	C18—C19—C20—C21	0.3 (6)
Br1—Cd1—Br4C—Br4A	68.0 (7)	C22—C19—C20—C21	178.6 (4)
Br3—Cd1—Br4C—Br4A	−180.0 (7)	C19—C20—C21—C16	0.1 (6)
Br4B—Cd1—Br4C—Br4A	−22.6 (5)	C17—C16—C21—C20	−0.3 (6)
Br4A—Cd1—Br4C—Br4B	22.6 (5)	S1—C16—C21—C20	179.8 (3)
Br2—Cd1—Br4C—Br4B	−44.5 (5)	C28—N2—C22—N1	−0.6 (4)
Br1—Cd1—Br4C—Br4B	90.6 (4)	C28—N2—C22—C19	−179.7 (3)
Br3—Cd1—Br4C—Br4B	−157.4 (4)	C23—N1—C22—N2	0.8 (4)
C1—S2—C2—C3	1.6 (4)	C23—N1—C22—C19	179.9 (3)
C1—S2—C2—C7	−178.1 (3)	C18—C19—C22—N2	9.0 (6)
C7—C2—C3—C4	−0.6 (6)	C20—C19—C22—N2	−169.2 (4)
S2—C2—C3—C4	179.7 (3)	C18—C19—C22—N1	−169.9 (4)
C2—C3—C4—C5	1.0 (6)	C20—C19—C22—N1	11.8 (6)
C3—C4—C5—C6	−0.9 (6)	C22—N1—C23—C28	−0.7 (4)
C3—C4—C5—C8	178.5 (3)	C22—N1—C23—C24	179.1 (4)
C4—C5—C6—C7	0.4 (6)	C28—C23—C24—C25	0.4 (6)
C8—C5—C6—C7	−179.0 (4)	N1—C23—C24—C25	−179.4 (4)
C5—C6—C7—C2	0.0 (6)	C23—C24—C25—C26	−0.1 (6)
C3—C2—C7—C6	0.1 (6)	C24—C25—C26—C27	−0.2 (7)
S2—C2—C7—C6	179.8 (3)	C25—C26—C27—C28	0.1 (6)
C9—N4—C8—N3	0.6 (4)	N1—C23—C28—C27	179.3 (3)
C9—N4—C8—C5	−178.4 (4)	C24—C23—C28—C27	−0.5 (6)
C10—N3—C8—N4	−0.8 (4)	N1—C23—C28—N2	0.3 (4)
C10—N3—C8—C5	178.3 (3)	C24—C23—C28—N2	−179.5 (4)
C6—C5—C8—N4	179.6 (4)	C26—C27—C28—C23	0.2 (6)
C4—C5—C8—N4	0.2 (6)	C26—C27—C28—N2	178.9 (4)
C6—C5—C8—N3	0.8 (6)	C22—N2—C28—C23	0.2 (4)
C4—C5—C8—N3	−178.6 (4)	C22—N2—C28—C27	−178.7 (4)

### Hydrogen-bond geometry (Å, °)

**Table:**

*D*—H···*A*	*D*—H	H···*A*	*D*···*A*	*D*—H···*A*
N1—H1N···Br3	0.79 (7)	2.51 (7)	3.272 (5)	164 (5)
N2—H2N···Br2^i^	0.81 (7)	2.50 (7)	3.267 (4)	160 (5)
N4—H4N···O1^ii^	0.83 (7)	1.88 (7)	2.679 (6)	161 (6)
C4—H4···O1^ii^	0.95	2.55	3.464 (8)	160

Symmetry codes:  (i) *x*, −*y*+1/2, *z*−1/2; (ii) *x*+1/2, *y*, −*z*+1/2.
